# Supplementary material for: Real-World Incidence of Febrile Neutropenia among Patients Treated with Single-Agent Amrubicin: Necessity of the Primary Prophylactic Administration of Granulocyte Colony-Stimulating Factor
Source: J Clin Med. 2021 Sep 17;10(18):4221. doi: 10.3390/jcm10184221 (PMC8464789; doi:10.3390/jcm10184221)
Supplement: Supplementary file 1 [file jcm-10-04221-s001.zip › jcm-1348502-supplementary.pdf]

Table S1. Administration of therapeutic G-CSF (*N* = 156).

	**Number**	**%**
Patients treated with G-CSF	112	72
Reasons for receiving G-CSF		
Neutropenia	65	42
Febrile neutropenia	47	30

Abbreviation: G-CSF, granulocyte colony-stimulating factor.

Table S2. Outcomes of febrile neutropenia (*N* = 47).

	**Number**	**%**
Antibiotic treatment	47	100
Treatment delay	18	38
Dose reduction	15	32
Transition to best supportive care	14	30

Abbreviation: PS, Eastern Cooperative Oncology Group performance status.
